# Influence of activation mode and aeration rate on ferritization efficiency, phase formation, and sediment stability in spent etching solution treatment

**DOI:** 10.1038/s41598-026-45599-7

**Published:** 2026-04-12

**Authors:** Gennadii Kochetov, Dmitry Samchenko, Yuliia Trach, Oles Lastivka

**Affiliations:** 1 Faculty of Engineering Systems and Ecology, Kyiv National University of Construction and Architecture, Kyiv, 03680 Ukraine; 2 Institute of Civil Engineering and Architecture, National University of Water and Environmental Engineering, Rivne, 33028 Ukraine; 3 Institute of Civil Engineering, Warsaw University of Life Sciences, Warsaw, 02-776 Poland; 4 Department of Technology of Building Structures and Products, Kyiv National University of Construction and Architecture, Kyiv, 03680 Ukraine

**Keywords:** Ferritization, Spent etching solutions, Activation methods, Iron-containing sediments, Chemistry, Engineering, Environmental sciences, Materials science

## Abstract

The growing volume of spent etching solutions generated by the metallurgical industry represents a significant environmental challenge due to their high acidity and high concentrations of dissolved iron. Developing efficient treatment methods that ensure both decontamination and resource recovery is a priority for sustainable industrial wastewater management. Ferritization is a process that converts dissolved iron into stable ferrite phases and thus offers an effective pathway for transforming hazardous waste into environmentally safe and technologically valuable materials. This study evaluates the ferritization of diluted sulfuric acid under three activation conditions: thermal, ultrasonic, and alternating magnetic field. Experiments were conducted with air oxygen bubbling rate of 0.02–0.06 dm³/s and reaction durations of 30–75 min. The resulting precipitates were characterized using X-ray diffraction to determine their phase composition, while leaching tests assessed the stability of the ferrite products in aqueous environments. The results show that thermal activation at 75 °C combined with the highest aeration rate significantly enhances the conversion of iron oxyhydroxides into magnetite (Fe₃O₄). Under optimal conditions, magnetite content approached 100%, iron removal reached 99.99%, and leaching of iron ions did not exceed 0.2 mg/dm³. Overall, the study demonstrates that ferritization is an efficient and environmentally viable method for treating spent pickling solutions while obtaining valuable iron-containing products.

## Introduction

Industrialization and the increasing scale of production in the metallurgical, mechanical engineering, chemical, and electroplating industries have led to a significant influx of heavy metal ions into the environment, primarily through wastewater discharges. Ions of lead (Pb²⁺), cadmium (Cd²⁺), zinc (Zn²⁺), copper (Cu²⁺), chromium (Cr⁶⁺/Cr³⁺), and nickel (Ni²⁺) are typical contaminants of industrial effluents. These ions are characterized by high toxicity, cumulative effects, and resistance to biodegradation^1,2^. The entry of such compounds into aquatic ecosystems poses a serious threat to both human health and aquatic organisms; therefore, their effective removal is one of the key objectives of modern water treatment technologies.

Among the main methods used for the removal of heavy metal ions from wastewater are chemical precipitation, ion exchange, membrane processes, electrochemical methods, and sorption^1,3, 4^. Despite its simplicity, chemical precipitation produces large volumes of sludge and is often ineffective at low metal concentrations. Membrane methods provide high efficiency but require significant energy input and frequent cleaning. Ion exchange demonstrates good selectivity but involves the use of regeneration reagents, which generate additional waste streams.

The sorption method, particularly when using natural materials, is considered a promising alternative due to its simplicity, relatively low cost, the possibility of multiple reuse of sorbents, and the wide availability of raw materials^5–7^. Over the past decade, interest in magnetic sorbents has grown rapidly because of their high efficiency in removing heavy metal ions from wastewater. The most common examples are iron oxide-based nanoparticles, such as magnetite (Fe₃O₄), maghemite (γ-Fe₂O₃), and hematite (α-Fe₂O₃), which combine magnetic and sorptive properties^8,9^. Additionally, iron oxyhydroxides-goethite (α-FeOOH), lepidocrocite (γ-FeOOH), and ferrihydrite - are actively studied due to their high specific surface area and reactive functional groups, which ensure strong affinity for Pb²⁺, Cd²⁺, Zn²⁺, and Cu²⁺ ions in wastewater^10^.

The chemical stability of magnetic sorbents, particularly their resistance to iron ion leaching, is a crucial parameter for their application in water treatment technologies. The loss of Fe²⁺/Fe³⁺ ions reduces the service life of the sorbent and deteriorates the quality of treated water^11^. Iron oxides such as hematite, maghemite, and magnetite are stable over a wide pH range, especially in neutral and mildly alkaline environments^12^. Hematite exhibits the highest stability due to its dense crystal lattice and extremely low solubility (K_sp_ ≈ 10⁻⁴³), while magnetite is less stable in acidic conditions (pH < 4), leading to the selective dissolution of Fe²⁺ ions^13,14^. Iron oxyhydroxides are metastable phases and more soluble under acidic conditions (pH 2–4), where they form hydrated iron complexes [Fe(H₂O)₆]²⁺/³⁺. Prolonged exposure may result in the dissolution of 20–40 mg/L of Fe³⁺^15,16^.

Low chemical stability of sorbents can cause secondary water contamination with iron, particle disintegration, and a decrease in sorption capacity during repeated use^17,18^. To improve material stability, protective coatings or mixed ferrites are often employed, with partial substitution of Fe by more stable cations such as Co, Ni, or Mn^19^.

Modern eco-technologies are increasingly focused on the utilization of industrial waste as a raw material for the synthesis of functional materials, particularly magnetic sorbents based on iron oxides. One of the most promising secondary resources is spent etching solutions, which contain a high concentration of Fe²⁺/Fe³⁺ ions^20,21^. Their use allows for the recycling of toxic components while reducing the production cost of sorbents.

For the synthesis of magnetic materials under laboratory conditions, several methods are commonly applied, including hydrothermal, solvothermal, ferritization, thermal decomposition, microemulsion, electrochemical, and microwave methods^22^. Among these, hydrophase ferritization is considered the most promising because it enables the recovery of secondary products from water treatment wastes^23^. The ferritization process typically occurs at temperatures above 75 °C, making it energy-intensive; however, alternative activation methods such as ultrasound and alternating magnetic fields can reduce energy consumption and improve particle morphology^24,25^.

The aeration rate is a key factor in the hydrophase ferritization process, as it affects the oxidation degree of Fe²⁺ to Fe³⁺, the phase composition of the sediment, particle size, and their magnetic and sorption properties^26–29^. Optimal aeration promotes the formation of magnetite (Fe₃O₄) with desired characteristics, while excessive or insufficient aeration may lead to the formation of maghemite (γ-Fe₂O₃) or unstable hydroxides.

Thus, spent etching solutions and other iron-containing liquid wastes represent a valuable raw material for environmentally oriented production of magnetic sorbents, combining the recycling of hazardous components with the generation of materials of high functional value. Based on the performed analysis and considering current challenges in the synthesis of stable magnetic sorbents, this study proposes a systematic approach to study the influence of technological parameters on the properties of ferrite sediment.

The scientific novelty of this research lies in the integrated approach to the synthesis of magnetic ferrite sorbents from spent etching solutions, involving controlled aeration rates and various activation methods of the reaction mixture. For the first time, the combined effect of aeration rate and activation method on the phase composition, sorption properties of the sediment has been systematically studied. Additionally, criteria for selecting stable ferrite phases suitable for multiple reuse in water treatment technologies have been developed, ensuring both waste recycling and the production of highly efficient magnetic sorbents.

The aim of the study is to obtain ferrite materials from spent etching solutions via the ferritization method under air–oxygen bubbling rates and different activation modes, for their subsequent use as magnetic sorbents in the purification of wastewater from heavy metal ions.

Research objectives:

- To determine the effect of air–oxygen bubbling rate and activation method of the ferritization reaction mixture on the efficiency of iron ion removal from etching solutions;
- To study the phase composition of ferritization sediment in order to assess their suitability as sorbents;
- To study the chemical stability of ferrite materials, particularly their resistance to iron ion leaching.

## Materials and methods

For the experimental studies, sediment were obtained as a result of the ferritization treatment of spent sulfuric acid etching solutions. The initial solution was collected from one of the Ukrainian industrial enterprises, where the concentration of ferrous ions (Fe_total_) was 145 g/dm³, FeSO_4_ 394 g/L, pH 1.21.

To ensure proper experimental conditions, the solution was diluted tenfold with tap water, reducing the iron ion concentration to 14.5 g/dm³. The pH value was adjusted to 10.5 using a 50% sodium hydroxide solution, and pH control was performed with an “Adwa” AD 1040 series pH meter.

Four series of experiments (A-D) on ferritization processing of exhausted etching solutions were carried out. In series C, the ferritization process was studied at a temperature of 20 °C and constant stirring of the solution with an overhead mixer at a speed of 250 rpm. Two alternative activation methods were used: alternating magnetic field and ultrasound were used in series of experiments A and B, respectively. The traditional method of thermal activation at 75 °C was studied in series of experiments D. Standard air aeration was used in the experiments. The oxygen content in the room air, measured with an AKT EZODO 7031 oximeter, was 20.2 ± 0.1%.

To aerate the reaction mixture with air oxygen at a controlled rate, a “Xilong” XL-008B laboratory compressor combined with an air distribution system was used. The air bubbling rate was measured using a “Tenmars Electronics” TM-4001/4002 anemometer. To measure the air aeration rate in the experiments, a Zyia LZM-15ZT-0.2–2 GPM rotameter was used (measurement accuracy ± 4%). The standard deviations for the aeration time were ± 2 s.

In addition to thermal activation, the effects of alternative activation methods - ultrasonic treatment and alternating magnetic field (AMF) activation were studied. For the ultrasonic treatment experiments, a square ultrasonic bath “TUN”, series 13, with a volume of 1.3 L was used. The bath is equipped with a transducer located at the bottom, which ensures stable and reproducible intensity of its effect on the solution. The ultrasonic power was 70 W at a frequency of 40 kHz. The reactor with the aeration system occupied about 50% of the bath volume and was positioned at its center. For AMF activation, a controlled rectangular pulse generator based on the ATmega328p microcontroller board was used. The parameters of the alternating magnetic field were controlled via a UTP3305C power supply connected through an RS-232 interface. The settings were as follows: magnetic induction amplitude – 0.1 T, pulse frequency – 1 Hz, interval between pulses – 100 ms, and pulse duration – 1000 ms.

The coil was constructed from copper wire with a diameter of 2.5 mm and equipped with a core made of electrical steel grade Eh0300. The magnetic field strength was measured using a Lake Shore F41 teslameter. Measurements were carried out at the center of the magnetic circuit gap, which ensured the uniformity of the magnetic field within the working volume.

All experiments were conducted in reactors with a working volume of 1.0 dm³. The laboratory setups and procedures for obtaining the sediment are described in detail in previous works: thermal activation^30^, AMF activation^31^, and ultrasonic treatment^32^. The technological parameters for obtaining ferrite sediment are summarized in Table 1.

Previous studies on ferritization processing of etching solutions allowed for the determination of optimal pH values, iron ion concentrations, and reaction durations. Since the influence of air-oxygen bubbling rate on the characteristics of the ferrite sediment remains insufficiently studied, in this work the bubbling rate was varied within the range of 0.02–0.06 dm³/s depending on the chosen activation method of the reaction mixture.

**Table 1:** Conditions for obtaining iron-containing sediments from exhausted etching solutions via ferritization.

Sample no.	The aeration rate of air oxygen, dm^3^/с	The time of the ferritization process, min	pH of the reaction mixture	Concentration Fe^2+^ ions, g/dm^3^
1	0.02	30	10.5	14.5
2	0.03	30		
3	0.04	30		
4	0.05	30		
5	0.06	30		
6	0.06	75		

After ferritization, the precipitate was thickened using a Micromed CM-5 centrifuge and subsequently dried for 24 h at 105 °C in an electric drying oven SNOL 67/350.

The phase analysis of the obtained sorbent materials was performed using X-ray diffraction (XRD) in a step-scan mode with Cu-Kα radiation on a Rigaku Ultima IV diffractometer. Sorbent powders were scanned within the 2θ range of 6–65°, using a step size of 0.05° and an exposure time of 2 s per point. The diffraction patterns were interpreted using the ICCD PDF2 + − 2003 database (The International Centre for Diffraction Data) and the Crystal Impact Match V.1.9a, Bonn, Germany^33^.

The residual concentration of iron ions in the solution after precipitate removal was determined spectrophotometrically using a Hach DR3900 spectrophotometer, with a measurement accuracy of 0.02–3.00 mg/dm³. A standard reagent set (No. 2105769) for total iron determination was used The analyses were performed three times for each sample.

To assess the chemical stability of the ferritized sorbent materials, leaching experiments were carried out to extract iron ions, followed by analysis of the eluates. After the leaching process, the residual iron concentration in each eluate was measured using the same spectrophotometer (Hach DR3900).

**Fig. 1:**
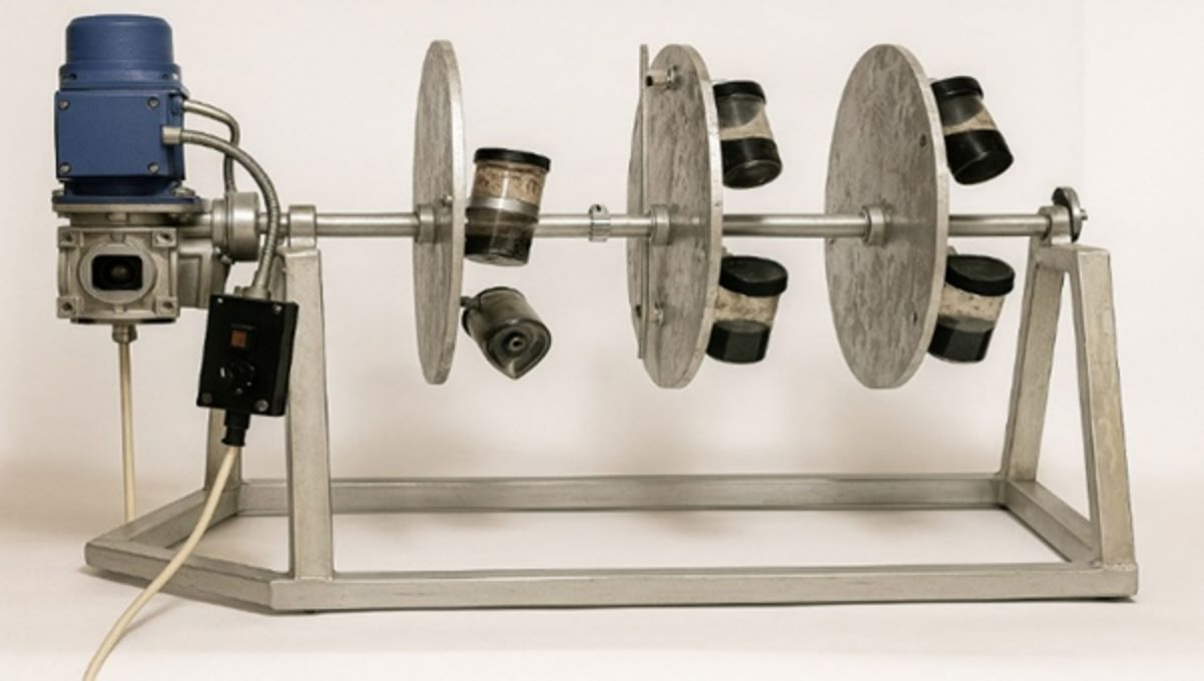
Experimental setup used for ferritization of spent etching solutions. Photograph taken by the authors.

Leaching was performed in polypropylene cylindrical containers with a nominal volume of 500 mL. The container volume was selected based on the dry mass of the sample (175 g) to minimize free space. The leaching process was carried out under static conditions, simulating the effect of atmospheric precipitation on sludge stored in open disposal sites. For gentle mixing of the samples with the eluate, a mechanical stirring apparatus with a rotation speed of 9 rpm was used. The schematic of the setup is presented in Fig. 1.

Dried ferritization precipitate samples were ground in a porcelain mortar, and sieving was conducted using sieves with a nominal mesh size of 4 mm.

To evaluate the effect of pH on the migration rate of heavy metal ions, experimental studies were conducted on iron leaching from the ferritized sediment. Distilled water with a neutral pH = 6.8 was used, while acidic and alkaline environments were simulated using:

- *acid rain model*: sulfuric acid solution (H₂SO₄, pH = 4.0), and
- *alkaline soil model*: sodium hydroxide solution (NaOH, pH = 9.0).

Tests were conducted on materials where at least 95% by mass consisted of particles smaller than 4 mm. If the proportion of larger particles exceeded 5% by mass, the coarse fraction was additionally ground. When the laboratory sample contained excess moisture and could not be ground or sieved, pre-drying was permitted.

The entire test sample that met the particle size criterion was not subjected to further drying. The dry residue mass was determined at a temperature of 105 °C ± 5 °C.

The moisture content coefficient (dry matter content) was calculated using Eq. (1):

1 $$\:DR=100\cdot\:\frac{{M}_{D}}{{M}_{W}},$$

where DR – dry matter content coefficient, %; M_D_ – mass of the test sample after drying, kg; M_W_ – mass of the test sample before drying, kg.

The moisture content coefficient was calculated using Eq. (2):

2 $$\:{M}_{C}=100\cdot\:\frac{\left({M}_{W}-{M}_{D}\right)}{{M}_{D}},$$

A test portion of the precipitate sample was prepared with a total mass (M_W_) containing 0.175 kg ± 0.005 kg of dry matter (M_D_), which was calculated using Eq. (3):

3 $$\:{M}_{W}=100\cdot\:\frac{{M}_{D}}{DR},$$

Leaching tests for iron ions from the iron-containing materials were carried out at room temperature (20 ± 5 °C) and different pH values.

The next step involved adding the required volume of leaching solution of varying pH values (L). The liquid-to-solid ratio (L/S) was maintained at 2 dm³/kg ± 2%, calculated according to Eq. (4). Proper mixing of solid and liquid phases was ensured:

4 $$\:L=\left(2-\frac{MC}{100}\right)\cdot\:{M}_{D},$$

where L – volume of leaching solution spent, L; MC – moisture content coefficient, %.

The test portion and leachate were hermetically sealed in the container and mounted on a rotating device for 24 h mixing. Before further analysis, sedimentation was performed to separate suspended particles from the leachate. Sedimentation was achieved by centrifugation at 10.000 rpm for 5 min. After phase separation, the chemical analysis of the leachate was conducted using spectrophotometry to determine the concentration of iron ions.

The analysis yielded the iron ion concentration in the leachate, expressed in mg/dm³. The final result represents the mass of leached components relative to the dry weight of the sample, expressed in mg/kg of dry matter.

The amount of a component leached from the material, based on the dry mass of the original sample, was calculated according to Eq. (5):

5 $$\:A={C}_{зал.}\cdot\:\left[\left(\frac{L}{{M}_{D}}\right)+\left(\frac{MC}{100}\right)\right],$$

where A – amount of leached component at L/S = 2 (dm³/kg of dry matter); C_e_– residual concentration of the component in the leachate, mg/dm³; L – volume of leaching solution spent, dm³; MC – moisture content coefficient, expressed as a percentage of dry mass; MD – dry mass of the test portion, kg.

The dispersion and error limits of experiments for determining residual concentrations of zinc ions after sorption were assessed according to method^34^ with a confidence probability of 0.95.

All quantitative measurements were performed in triplicate (*n* = 3), and the results are reported as mean ± standard deviation (SD). The standard deviation was selected as the primary measure of variability because it reflects the dispersion of individual experimental values around the mean and thus characterizes the intrinsic reproducibility of the ferritization process under identical conditions.

## Results and discussion

A comprehensive approach to the treatment of spent etching solutions was aimed not only at achieving purification levels that meet current regulatory standards for the reuse of treated water in electroplating operations, but also at obtaining water treatment sediment whose quality and environmental safety enable their subsequent use in the production of sorbents. This approach makes it possible to obtain cost-effective commercial products from secondary raw materials.

### Study of the influence of air–oxygen bubbling rate of the reaction mixture in the ferritization process on the efficiency of iron ion removal from etching solutions under different activation methods

**Fig. 2:**
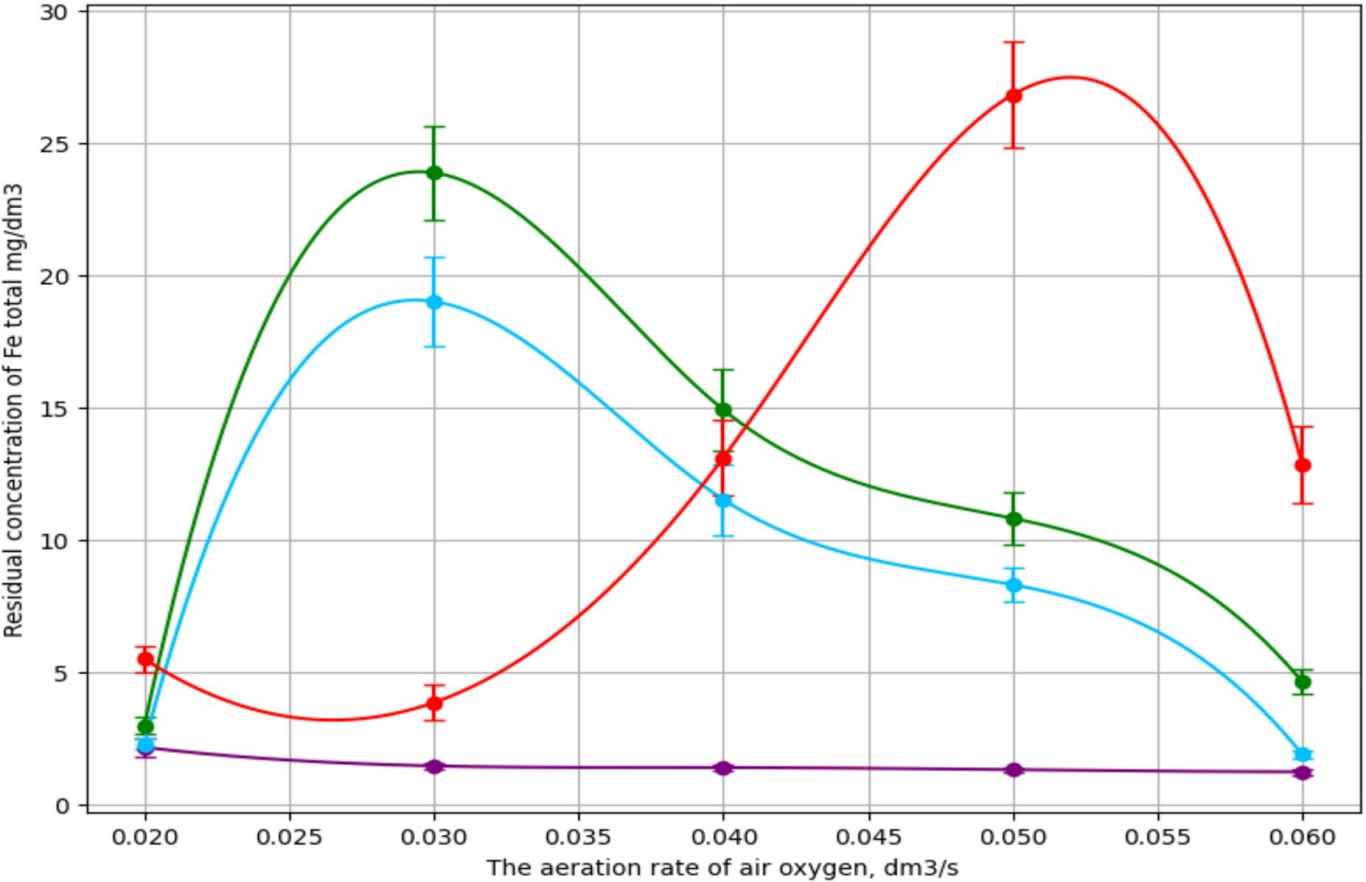
Removal of iron ions from exhausted etching solutions; by ferritization at 20 °C - 1, using activation of the reaction mixture: AMF - 2, ultrasonic treatment -3, thermal - 4.

The results of iron ion removal under different activation methods and air-oxygen bubbling rates are presented in Fig. 2. Measurements of total concentration for iron ions at each point were performed three times. The data indicate that activation methods and bubbling intensity significantly affect the efficiency of iron ion removal, most likely due to phase transformations occurring during ferritization.

When the reaction mixture was activated by alternating magnetic field (AMF) or ultrasonic treatment, a similar dependence of residual iron ion concentration on bubbling rate was observed. Based on previous studies, this can be explained by the formation of intermediate hydroxide and oxyhydroxide phases of iron, which, with increased oxygen supply, transform into more stable oxide compounds. For instance, increasing the bubbling rate from 0.03 to 0.06 dm³/s insignificantly improved the removal efficiency from 99.84% and 99.87% to 99.96% and 99.98% for ultrasonic and electromagnetic activation, respectively.

A different pattern was observed during ferritization at 20 °C. The lowest degree of iron ion removal was recorded at a bubbling rate of 0.05 dm³/s, with a purification efficiency of 99.82%. This may be attributed to slower phase transformation of hydroxides and oxyhydroxides in the alkaline medium at lower temperatures.

The highest removal efficiency was achieved under thermal ferritization at 75 °C in the entire range of bubbling rates. Under these conditions, even at relatively low oxygen input, stable oxide phases were formed. The total iron concentration in the treated solution ranged between 1.14 and 2.12 mg/dm³, corresponding to a purification efficiency of 99.99%.

However, despite the high degree of purification, the treated water did not meet the reuse standards for galvanic rinsing operations in terms of iron ion content (C_Fe_ ≤ 0.3 mg/dm³). Nevertheless, the water quality complied with discharge standards for municipal sewer systems (C_Fe_ ≤ 2.5 mg/dm³), including those in Kyiv^35,36^.

Further experiments aimed to enhance purification quality by increasing ferritization time at the optimal bubbling rate of 0.06 dm³/s. The process was studied under different activation methods and durations (15–75 min), while maintaining constant iron ion concentration and pH.

**Fig. 3:**
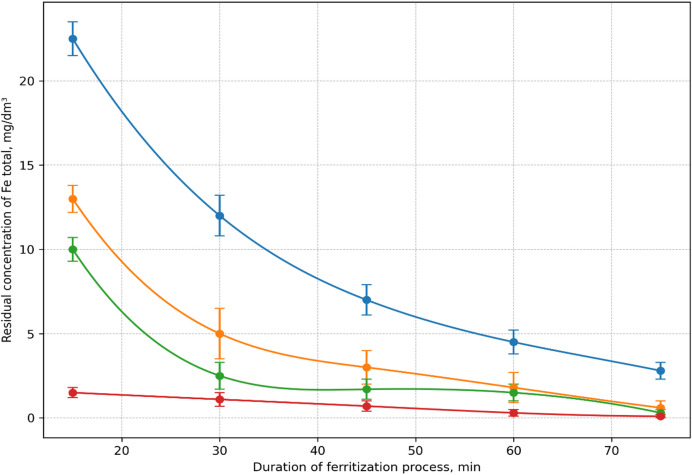
Kinetics of iron ions removal from exhausted etching solutions; by ferritization at 20 °C − 1, using activation of the reaction mixture: AMF − 2, ultrasonic treatment − 3, thermal – 4.

The results (Fig. 3) confirmed that, regardless of activation method, residual iron content decreases with longer ferritization time. The lowest concentrations were recorded after 75 min of processing. Specifically, under thermal ferritization at 75 °C, the total iron concentration decreased to 0.08 mg/dm³, corresponding to 99.99% purification efficiency. For AMF activation and ultrasonic treatment, the concentrations were 0.162 and 0.276 mg/dm³, respectively, with the same purification degree of 99.99%.

Only at a process duration of 75 min and activation by temperature of 75 °C, alternating magnetic field or ultrasound was it possible to achieve iron concentrations meeting permissible limits (MPC) for both reuse in galvanic operations and discharge into municipal sewer systems. The obtained results were further substantiated through X-ray phase analysis of the ferritization sediment, confirming the dependence of purification efficiency on process parameters.

### Structural analysis of ferritization sediment

The phase composition of iron-containing ferritization sediment obtained under different activation methods and oxygen aeration rates of the reaction mixture was studied. Considering the known mechanism of the ferritization process, a complex qualitative and quantitative phase composition of the sediment was expected, as they may contain various modifications of iron oxides and oxyhydroxides.

**Fig. 4:**
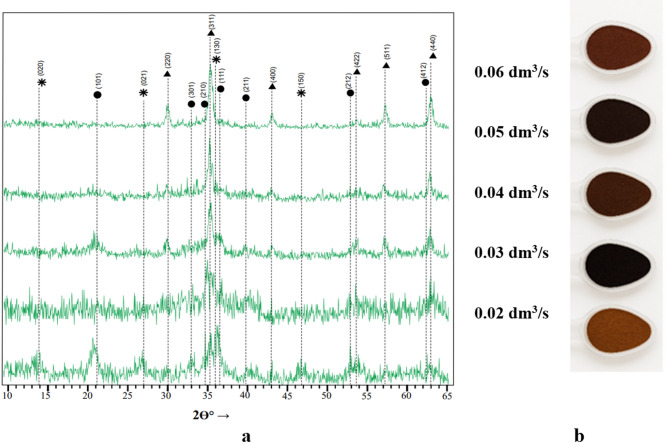
Diffraction patterns (**a**) and general view (**b**) of sediments obtained by ferritization of exhausted etching solutions using AMF activation of the reaction mixture.

The X-ray diffraction patterns of the sediment obtained under alternating magnetic field (AMF) activation, as well as the corresponding phase compositions determined from the diffraction data, are presented in Fig. 4a, while images of the samples are shown in Fig. 4b. The results indicate that all studied samples contain exclusively ferromagnetic crystalline phases. In particular, the ferritization sediment revealed the presence of chemically stable ferromagnetic magnetite (Fe₃O₄) with a lattice parameter of 8.36 Å.

In addition to magnetite, other solid-phase products of the ferritization reaction were detected, such as lepidocrocite (γ-FeOOH) and feroxyhyte (δ-FeOOH) with lattice parameters of a = 3.85 Å and 2.95 Å, respectively. It should be noted that the oxyhydroxide phases are relatively unstable in alkaline medium environments. In contrast, magnetite is resistant not only to water but also to diluted aqueous solutions of strong mineral acids and bases due to its spinel-type crystal structure.

Analysis of the diffraction data showed that the sample obtained at an aeration rate of 0.06 dm³/s exhibited a higher degree of crystallinity compared with the other samples. This was confirmed by the increase in intensity and the reduction in the width of the diffraction peaks at 2θ = 35.4° (reflection index 311). In other cases, the diffraction peaks were less intense and broader, indicating a lower degree of structural ordering.

Visual analysis of dried ferritization sediment obtained under AMF activation at different aeration rates revealed a clear dependence of color on aeration intensity (Fig. 4b). Light brown shades indicated the predominance of oxyhydroxide phases of iron, while darker colors corresponded to samples with a higher content of magnetite. This relationship is practically significant, as the formation of magnetite enhances the chemical stability of the sediment and imparts a dark color, expanding their potential for further applications, such as pigment materials. It should be noted that at a high level of aeration, along with magnetite, a small amount of X-ray amorphous iron hydroxide Fe(OH)₃ may form. Iron hydroxide has a brown color, and its presence in the ferritization precipitate affects its overall coloration.

Thus, the obtained ferritization sediments contain both oxyhydroxide and oxide phases of iron; the ratio of these phases determined by the process parameters, primarily the oxygen aeration rate.

Quantitative phase analysis results (Table 2) demonstrated that increasing the aeration rate of the reaction mixture leads to a higher magnetite content in the sediment, resulting from the sequential phase transformation: ɣ-FeOOH → δ-FeOOH → Fe₃O₄^37^.

**Table 2:** Phase composition of sediments obtained by ferritization of exhausted etching solutions using AMF activation.

Sample no.	Duration of ferritization process, min	The aeration rate, dm^3^/s	Phase content, %		
			ɣ-FeOOH	δ-FeOOH	Fe_3_O_4_
А-1	30	0.02	27.5	39.2	33.3
А-2	30	0.03	9.7	69.6	20.7
А-3	30	0.04	–	61.3	38.7
А-4	30	0.05	–	31.7	68.3
А-5	30	0.06	–	19.2	80.8
А-6	75	0.06	–	–	100

The results demonstrate that under maximum aeration and AMF activation, the solid products consisted of feroxyhyte (19.2%) and magnetite (80.8%). At aeration rate of 0.06 dm³/s and a process duration of 75 min, magnetite was the dominant phase. Its formation is attributed to the rapid generation of hydroxide and oxyhydroxide species of iron, which subsequently transform into the spinel structure of magnetite. The key stage of this process is reaction of disproportionation of polyvalent iron compounds, during which less stable phases are converted into the chemically stable magnetic structure of Fe₃O₄.

**Fig. 5:**
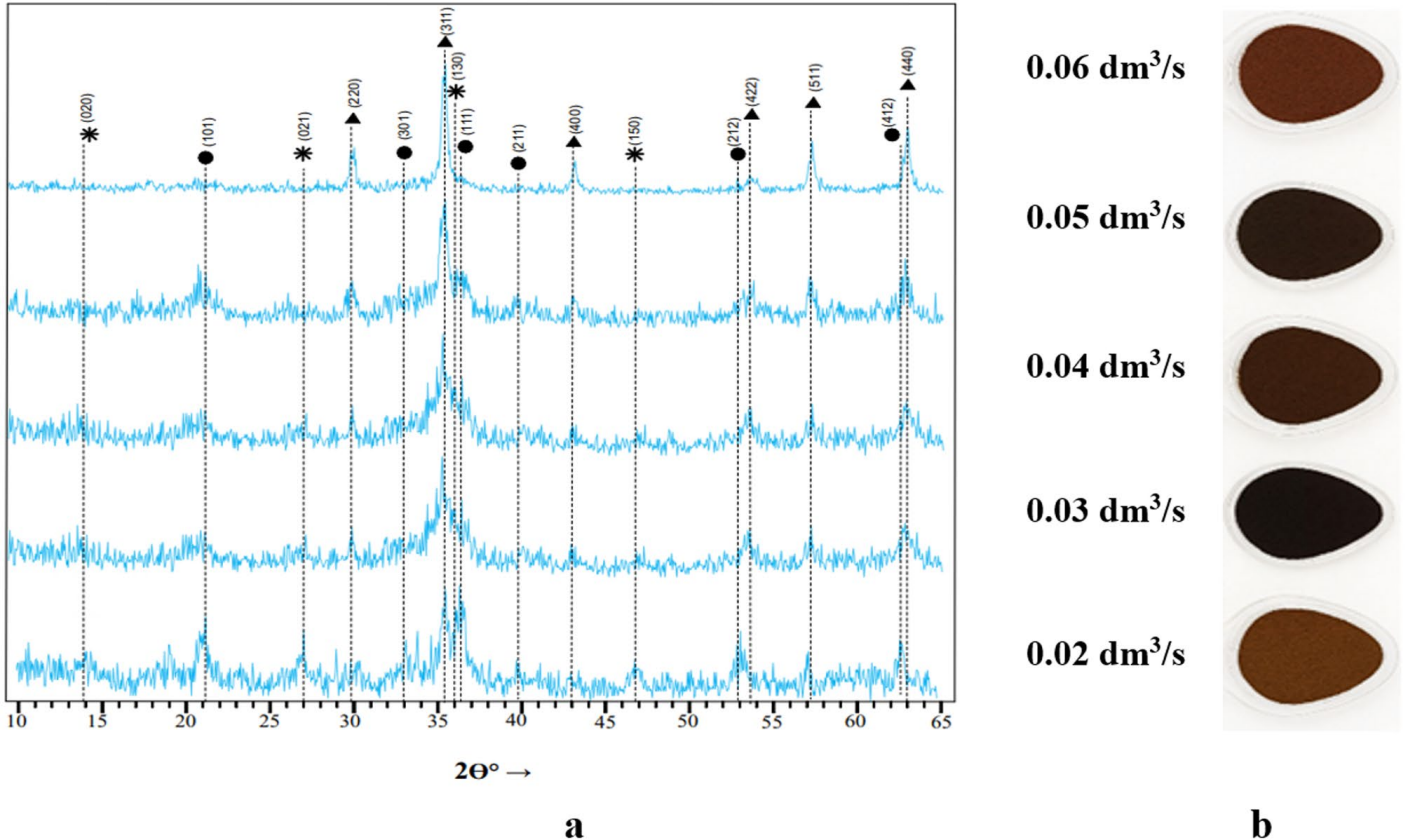
Diffraction patterns (**a**) and general view (**b**) of sediments obtained by ferritization of exhausted etching solutions using ultrasound activation of the reaction mixture.

The X-ray diffraction analysis of ferritization sediment obtained under ultrasonic activation (Fig. 5a) also confirmed a strong influence of aeration rate on the phase composition of the products. The phases γ-FeOOH, δ-FeOOH, and Fe₃O₄ were identified in the studied samples. Visual analysis of dried sediment obtained at different aeration rates (Fig. 5b) showed corresponding color changes: lighter shades indicated the predominance of oxyhydroxide phases, while darker colors corresponded to magnetite formation. These trends were consistent with those observed under AMF activation, indicating similar phase formation mechanisms under different energy activation methods. The intensification of ferritization process activation during ultrasonic treatment occurs primarily due to cavitation. The energy of ultrasonic waves propagating in the liquid is expended on the formation of cavitation bubbles, the generation of microflows, and local heating of the liquid. This promotes intensive mixing and enhances the formation of the solid phase throughout the entire volume of the reaction mixture. In turn, the magnetic field significantly reduces the activation energy of the ferritization reaction and accelerates its progress at room temperature.

According to Table 3, ultrasonic activation of the reaction mixture slows down phase transformations compared with AMF activation. This may be explained by the higher ability of the alternating magnetic field to initiate the stepwise formation of iron-containing compounds in solution compared to the insufficient intensity of the ultrasound effect to induce similar reactions. It should be emphasized that the optimal technological parameters of activation by an alternating magnetic field-specifically magnetic field strength, frequency range, treatment duration, and temperature conditions - were previously determined and experimentally substantiated in Kochetov et al.^31^. In that study, a systematic parametric optimization was carried out, which made it possible to establish an activation regime that ensures an accelerated progression of phase transformations and reproducible formation of the target iron-containing phases.

Nevertheless, under the highest aeration rate, the final phase products formed during ultrasonic activation were also feroxyhyte (20.7%) and magnetite (79.3%). These results closely match those obtained for AMF activation (Table 3), confirming the reproducibility of the ferritization process irrespective of the type of external energy influence.

**Table 3:** Phase composition of sediments obtained by ferritization of exhausted etching solutions using ultrasound activation.

Sample no.	Duration of ferritization process, min	The aeration rate, dm^3^/s	Phase content, %		
			ɣ-FeOOH	δ-FeOOH	Fe_3_O_4_
В-1	30	0.02	30.2	40.1	29.6
В-2	30	0.03	21.9	56.7	21.4
В-3	30	0.04	4.3	64.2	31.5
В-4	30	0.05	–	52.3	47.7
В-5	30	0.06	–	20.7	79.3
В-6	75	0.06	–	16.8	83.2

**Fig. 6:**
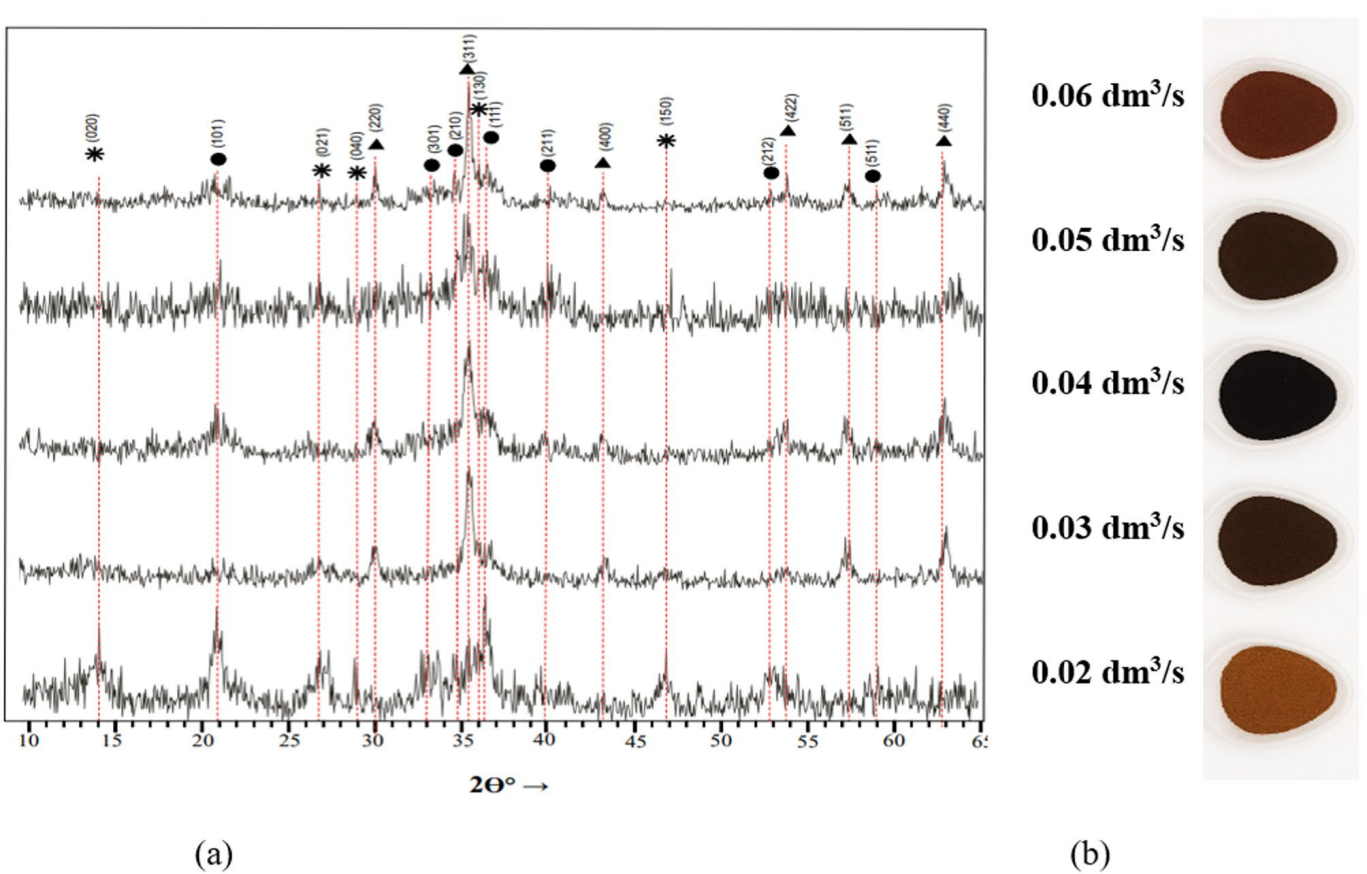
Diffraction patterns (**a**) and general view (**b**) of sediments obtained by ferritization of exhausted etching solutions using AMF activation at 20 °С.

The X-ray diffraction analysis of ferritization sediment obtained at 20 °C (Fig. 6a) revealed the presence of γ-FeOOH, δ-FeOOH, and Fe₃O₄ phases. The sample produced at an aeration rate of 0.02 dm³/s exhibited distinct peaks corresponding to intermediate compounds γ-FeOOH and δ-FeOOH. The broadening of these peaks indicated a low degree of crystallinity. Increasing the aeration rate to 0.05 dm³/s promoted the formation of a larger amount of intermediate phases, suggesting active phase transformations, though crystallinity remained lower than under magnetite-forming conditions.

Visual analysis of dried sediment obtained at 20 °C under different aeration rates (Fig. 6b) confirmed these trends. The color of the samples strongly depended on oxygen supply intensity: those synthesized at low aeration rates were yellowish due to the predominance of oxyhydroxide phases. Unlike the samples obtained under AMF or ultrasonic activation, these sediment exhibited greater color diversity, highlighting the influence of temperature and activation method on phase formation.

Quantitative phase composition data (Table 4) for the 20 °C ferritization process confirmed the predominance of chemically unstable γ-FeOOH and δ-FeOOH phases (> 50%) regardless of aeration rate. The sample obtained at 0.06 dm³/s contained lepidocrocite (2.4%), feroxyhyte (34.2%), and magnetite (63.4%). Thus, higher aeration rates promote the formation of more stable magnetite and reduce the proportion content of intermediate oxyhydroxides, improving the chemical stability of the sediments and their suitability for further utilization.

**Table 4:** Phase composition of the precipitates obtained during ferritization activation at 20 °C depending on the aeration rate and process duration.

Sample no.	Duration of ferritization process, min	The aeration rate, dm^3^/s	Phase content, %		
			ɣ-FeOOH	δ-FeOOH	Fe_3_O_4_
С-1	30	0.02	25.9	74.1	–
С-2		0.03	17.7	25.8	56.4
С-3		0.04	5.9	43.5	50.6
С-4		0.05	13.9	35.0	51.2
С-5		0.06	2.4	34.2	63.4
С-6	75	0.06	–	24.8	75.2

**Fig. 7:**
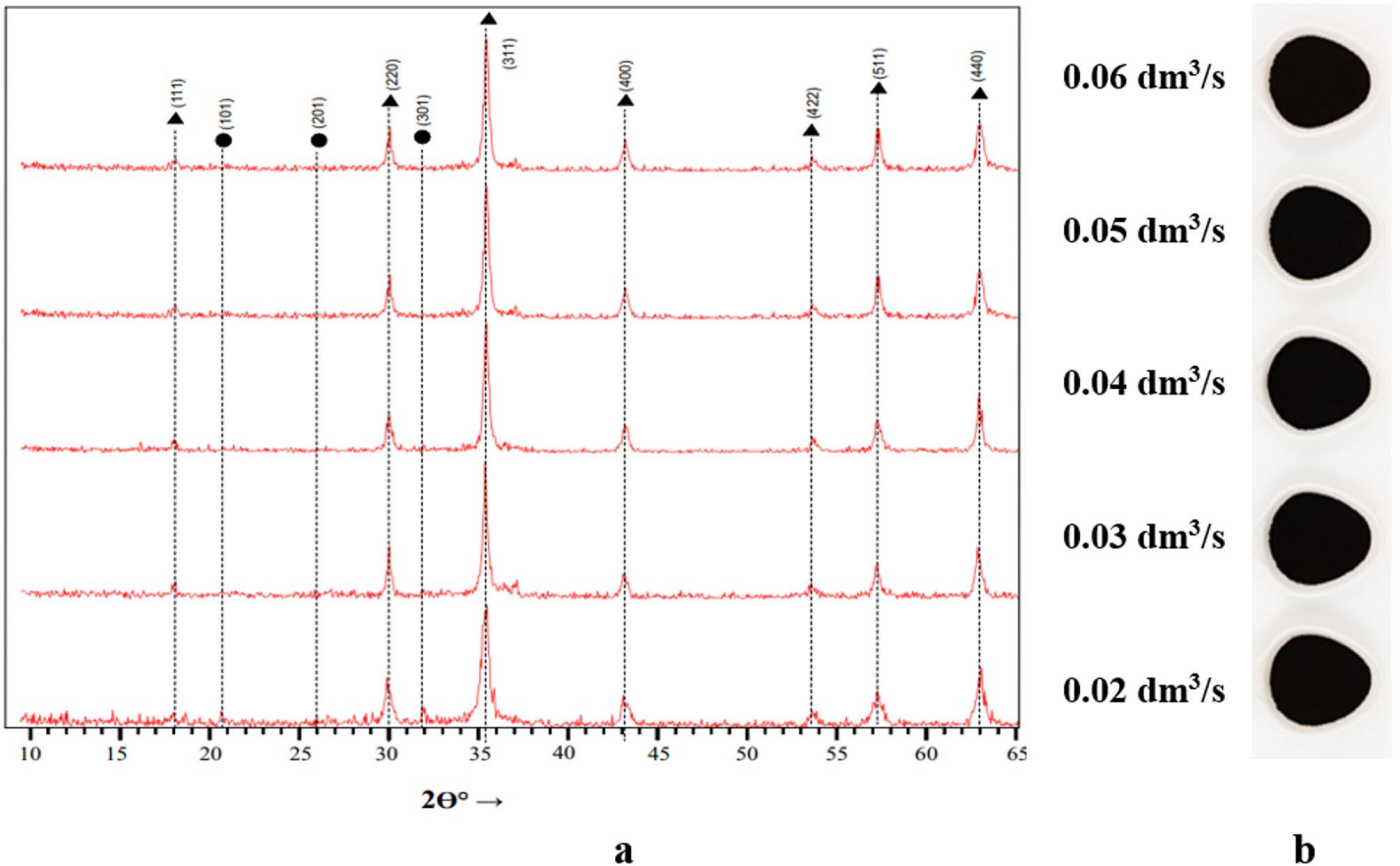
Diffraction patterns (**a**) and general view (**b**) of sediments obtained by ferritization of exhausted etching solutions using thermal activation of the reaction mixture.

Qualitative analysis of the thermally activated ferritization sediments demonstrated high crystallinity (Fig. 7a). The XRD pattern of the sample obtained at an aeration rate of 0.02 dm³/s showed reflections at 2θ = 21.1° and 31.9°, corresponding to the δ-FeOOH phase with reflection indices (101) and (301). Other samples exhibited have dominant peak at 2θ = 35.4° (index 311) characteristic of belonging to Fe₃O₄. This confirmed that magnetite is the main phase.

Quantitative phase analysis (Table 5) of the ferritization products obtained at thermal activation confirmed the formation of a maximum amount of chemically stable magnetite. For most samples 100% Fe₃O₄ content was recorded regardless of aeration rate, except for the lowest rate (0.02 dm³/s), where 7.5% δ-FeOOH was still detected. These results align with the chemical analysis of treated wastewater (Figs. 2 and 3) obtained after precipitate removal. Higher residual iron concentrations are observed when ferritization precipitates possess a predominantly amorphous structure. This phenomenon is attributed to the predominant role of the crystallization of insoluble compounds, primarily magnetite, in the removal of iron ions from spent etching solutions. The solubility product of magnetite in a neutral medium is extremely low (Ksp < 10⁻⁵⁰), which ensures efficient precipitation of iron.

**Table 5:** Phase composition of sediments obtained by ferritization of exhausted etching solutions using thermal activation.

Sample no.	Duration of ferritization process, min	The aeration rate, dm^3^/s	Phase content, %		
			ɣ-FeOOH	δ-FeOOH	Fe_3_O_4_
D-1	30	0.02	–	7.5	92.5
D-2	30	0.03	–	–	100
D-3	30	0.04	–	–	100
D-4	30	0.05	–	–	100
D-5	30	0.06	–	–	100
D-6	75	0.06	–	–	100

Visual inspection of dried sediment obtained under thermal activation at 75 °C (Fig. 7b) showed that all samples had a uniform black color, unaffected by changes in aeration rate. The color diversity indicates magnetite dominance in the phase composition under all tested conditions.

Thus, three activation methods were investigated. Comparing these methods, it can be noted that the thermal approach has a slight advantage in terms of efficiency and practical feasibility; however, the ultrasonic and magnetic methods are significantly more energy-efficient than the thermal one. This is confirmed by calculations of electricity consumption performed for each activation method.

Thermal ferritization requires 0.140 kWh to heat 0.5 dm³, taking into account the heater efficiency (90%), the operation of an 8 W compressor, as well as additional energy consumption to maintain the required temperature of the reaction mixture. Ultrasonic treatment of the reaction mixture consumes 0.078 kWh when using a 70 W ultrasonic bath. The energy consumption for activation by a pulsed magnetic field is 0.113 kWh. When the ferritization process is carried out at room temperature (20 °C), the energy consumption is only 0.008 kWh and is associated solely with the operation of the 8 W compressor. The comparative energy consumption of the ferritization process under different activation methods is presented in Table 6.

**Table 6:** Energy consumption during the ferritization process.

No.	Activation method	Electricity consumption, kWh	Relative energy consumption, %
1	Thermal ferritization	0.140	100
2	Ultrasonic treatment	0.078	55.7
3	Pulsed magnetic field (PMF)	0.113	80.7
4	Ferritization at 20 °C	0.008	5.7

In summary, it has been established that combining an optimal aeration rate with sufficient process duration ensures the formation of magnetite as the dominant phase in ferritization sediment. This determines their high crystallinity, chemical stability, and potential applicability as secondary raw materials.

### Results of the study on the chemical stability of iron-containing materials

To evaluate the chemical stability of the sorption materials via ion leaching, samples of ferritization sediment differing in quantitative and qualitative phase composition were selected. This approach allowed establishing the dependence of sorption properties on the structural characteristics of the ferritization products. The experiments involved samples with varying ratios of the main phases-magnetite (Fe₃O₄), maghemite (γ-Fe₂O₃), and iron oxyhydroxides (α-FeOOH, δ-FeOOH). The characteristics of the studied samples are presented in Table 7.

**Table 7:** Phase composition of sediments.

Sample no.	Phase content, %		
	ɣ-FeOOH	δ-FeOOH	Fe_3_O_4_
С−1	25.9	74.1	–
В−1	30.2	40.1	29.6
А−3	–	61.3	38.7
А−4	–	31.7	68.3
D−5	–	–	100

The study envisaged the potential use of ferritization sediment as sorbents in contact with aqueous media. Additionally, the possibility of their environmentally safe storage in open areas without risk of environmental contamination was considered.

To assess the chemical stability of the ferrite sediment, the leaching of iron ions was studied at different pH values (4.0, 6.8, and 9.0). The obtained results (Table 8) indicate that the leaching intensity strongly depends on the phase composition of the sediment, the conditions of their formation, and the method of activation of the reaction mixture. In particular, increased leaching was observed for sample C-1, which contained a significant amount of ferroxyhydrite (δ-FeOOH) - an unstable phase both in acidic and alkaline media. The content of this phase correlates directly with the increased concentration of iron ions in the aqueous solution.

Conversely, samples consisting of 100% magnetite exhibited high stability: the residual concentration of iron ions in the eluates did not exceed the regulatory limits established by DSanPiN 2.2.4-171-10 and Directive 98/83/EC for drinking water, where the maximum allowable concentration is 0.2 mg/dm³, irrespective of pH. Sediments obtained by thermal and AMF activation demonstrated reliable fixation of iron ions within the chemically stable inverse spinel structure of magnetite. In the process of ferritization, iron hydroxides are transformed into oxyhydroxides of various structural modifications according to reaction:

6 $${\text{2 Fe}}{\left( {{\mathrm{OH}}} \right)_{\mathrm{2}}}+{\text{ }}0.{\mathrm{5}}{{\mathrm{O}}_{\mathrm{2}}}\,=\,{\mathrm{2FeOOH}}\,+\,{{\mathrm{H}}_{\mathrm{2}}}{\mathrm{O}}$$

The mechanism of magnetite formation is obviously associated with the transformation of oxyhydroxides according to the following reaction:

7 $${\mathrm{Fe}}{\left( {{\mathrm{OH}}} \right)_{\mathrm{2}}}+\delta - {\mathrm{FeOOH}} \to {\mathrm{F}}{{\mathrm{e}}_{\mathrm{3}}}{{\mathrm{O}}_{\mathrm{4}}}\,+\,{\mathrm{2}}{{\mathrm{H}}_{\mathrm{2}}}{\mathrm{O}}$$

This confirms the high chemical stability of the obtained sediment and their environmental safety of the obtained sediment for potential practical applications.

Samples enriched with δ-FeOOH demonstrated enhanced iron ion leaching, leading to exceedance of the regulatory limits in aqueous media. Therefore, such materials cannot be recommended as sorbents in water treatment processes due to insufficient stability upon contact with aqueous systems.

**Table 8:** Leaching of iron ions from sediments.

Series of experiments	pH value of initial solution	Sample no.	Concentration of leached iron ions	
			А, mg/kg	С_end_, mg/dm^3^
1	9.0	C − 1	7.35	3.66
2	9.0	B − 1	3.12	1.54
3	9.0	А − 3	1.38	0.68
4	9.0	А − 4	0.43	0,0.21
5	9.0	D − 5	n.d.	n.d.
6	6.8	C − 1	2.45	1.21
7	6.8	B − 1	0.92	0.45
8	6.8	А − 3	0.45	0.22
9	6.8	А − 4	0.16	0.08
10	6.8	D − 5	n.d.	n.d.
11	4.0	C − 1	30.16	15.06
12	4.0	B − 1	13.17	6.57
13	4.0	А − 3	4.96	2.48
14	4.0	А − 4	3.67	1.82
15	4.0	D − 5	0.20	0.10

n.d.‒ beyond the threshold sensitivity of the measuring device 0.01 mg/dm^3^.

Further results on the application of ferritization products as sorption materials are reported by the authors^32^. The study discusses the effect of the phase composition of iron-containing samples (Table 6) on their sorption capacity for zinc ion removal during the treatment of rinse wastewater. The sorption activity of magnetic ferrite materials can be restored via chemical or electrochemical regeneration, allowing their repeated use without significant loss of efficiency^38,39^. Spent magnetic sorbents can be utilized as a component of alkaline construction materials, which additionally enhances the chemical stability of heavy metals within the material structure^40^.

Prospects for using chemically resistant ferritization products as magnetic sorbents for the removal of Zn²⁺ ions from industrial wastewater were examined in our previous work. The research conducted in this study makes it possible to synthesize such sorbents under the optimised conditions identified as most favourable for their production.

## Conclusions

The results of this study demonstrate that both activation mode of the reaction mixture and air oxygen bubbling rate have a significant influence on the efficiency of ferritization treatment for spent sulfuric acid etching solutions. It was established that higher aeration intensities accelerate the transformation of intermediate oxyhydroxides (γ-FeOOH and δ-FeOOH) into stable magnetite (Fe₃O₄), while the choice of activation method determines the rate and completeness of these phase transitions. The studied phase transformations significantly affect iron-ion removal efficiency.

Extending the ferritization duration to 75 min at a bubbling rate of 0.06 dm³/s resulted in the lowest residual concentrations of iron ions: 0.08, 0.16 and 0.27 mg/dm³ for thermal, AMF and ultrasonic activation, respectively. These values meet the regulatory standards for both reuse in galvanic rinsing processes and discharge into municipal sewer systems. XRD analysis confirmed that optimal conditions (75 °C, 0.06 dm³/s, 75 min) lead to the formation of a fully magnetite-based product (100% Fe₃O₄) with high crystallinity and uniform morphology. Among the tested activation approaches, thermal activation at 75 °C proved to be the most effective, ensuring iron-ion removal efficiencies of 99.99% over the full range of bubbling rates.

Chemical stability tests showed that magnetite-rich sediment exhibit minimal leaching of iron ions (< 0.2 mg/dm³ within pH 4.0–9.0), whereas materials containing δ-FeOOH demonstrate significantly higher iron release, limiting their suitability for sorption applications. The findings indicate that ferritization under optimized conditions yields chemically stable, environmentally safe iron-oxide materials that can be reused as effective magnetic sorbents, particularly for heavy-metal removal from industrial wastewater.

## Data Availability

All data generated or analyzed during this study are included in this published article.
